# A poly(dimethylsiloxane)-based solid-phase microchip platform for dual detection of Pseudorabies virus gD and gE antibodies

**DOI:** 10.3389/fcimb.2022.912108

**Published:** 2022-07-26

**Authors:** Jiaojiao Pan, Yufang Li, Tongyan Wang, Jingfeng Chang, Liying Hao, Junjie Chen, Wuping Peng, Junhua Deng, Baicheng Huang, Kegong Tian

**Affiliations:** ^1^ Luoyang Putai Biotech Co., Ltd., Luoyang, China; ^2^ Luoyang Zhongke Biochip Technology Co., Ltd., Luoyang, China; ^3^ National Research Center for Veterinary Medicine, Luoyang, China; ^4^ Department of Statistical Science, University College London, London, United Kingdom

**Keywords:** Pseudorabies virus, poly(dimethylsiloxane), protein chip, dual detection, clinical evaluation

## Abstract

Pseudorabies caused by pseudorabies virus (PRV) infection is still a major disease affecting the pig industry; its eradication depends on effective vaccination and antibody (Ab) detection. For a more rapid and accurate PRV detection method that is suitable for clinical application, here, we established a poly(dimethylsiloxane)-based (efficient removal of non-specific binding) solid-phase protein chip platform (blocking ELISA) for dual detection of PRV gD and gE Abs. The purified gD and gE proteins expressed in baculovirus were coated into the highly hydrophobic nanomembrane by an automatic spotter, and the gray values measured by a scanner were used for the S/N (sample/negative) value calculation (gD and gE Abs standard, positive: S/N value ≤0.6; negative: S/N value >0.7; suspicious: 0.6 < S/N ≤ 0.7). The method showed an equal sensitivity in the gD Ab test of immunized pig serum samples compared to the neutralization test and higher sensitivity in the gE Ab test compared to the commercial gE Ab detection kit. In the clinical evaluation, we found an agreement of 100% (122/122) in the gD Ab detection compared to the neutralization test and an agreement of 97.5% (119/122) in the gE Ab detection compared to the commercial PRV gE Ab detection kit. In summary, the protein chip platform for dual detection of PRV gD and gE Abs showed high sensitivity and specificity, which is suitable for PRV immune efficacy evaluation and epidemic monitoring.

## Introduction

Pseudorabies (PR) is caused by the infection of an alpha-herpesvirus Pseudorabies virus (PRV) (Pomeranz et al., 2005). The DNA genome of PRV is approximately 145 kb in size, containing almost 70 open reading frames (ORFs) that encode 70–100 viral proteins (Tan et al., 2017).

The herpesvirus PRV has a broad host range, which is known to cause acute fatal disease in a variety of mammals (Zhang et al., 2015; Wang et al., 2018; He et al., 2019). The PRV infection may lead to acute symptoms and death in pigs (Pomeranz et al., 2005), resulting in heavy economic losses in the pig industry.

PRV gE was critical for PRV virulence (Zhao et al., 2020); the gE-targeted ELISA has superiority in the differentiation of vaccinated and wild-type virus-infected animals (Schmitt et al., 1991; White et al., 1996). PRV gD is required for virus penetration (Peeters et al., 1992), and it is the most efficacious virus-neutralizing Ab target of PRV (Eloit et al., 1988; Marchioli et al., 1988; Mukamoto et al., 1991), suggesting that gD is an important indicator of immune protection and a suitable evaluative target for immune efficacy in diagnosis.

The emerging virulent PRV strains have caused severe PR in the vaccinated pigs in China since late 2011 (Yu et al., 2014; Gu et al., 2015). The PRV vaccines of classical strains only provide limited protection to the new-emerging PR (Yu et al., 2014). Therefore, it is urgent to develop a more rapid and accurate PRV detection method suitable for clinical application for the assessment of neutralizing Ab of emerging virulent PRV strains and differentiation between vaccine and wild-type strains.

Clinically, the methods in virus diagnosis include the liquid phase-based fluorescent microsphere immunoassay (Clavijo et al., 2006; Watson et al., 2009), colloidal-gold assay (Huang et al., 2020; Yang et al., 2020; Bai et al., 2021), and microarray-based detection (Wang et al., 2002; Rosenstierne et al., 2014; De Giorgi et al., 2019). For portable point-of-care (PoC) platforms, microarray technology allows fast, easy, and parallel detection of multiple addressable elements in a single experiment (Zhu and Snyder, 2003). The uniform and high-throughput coating of the target proteins in the membrane using an automatic spotter makes the detection more objective and conducive to mass production (Meade et al., 2017; Nakajima et al., 2018). Until now, no microchip platform for dual detection of PRV gD and gE has been reported.

Here, we established a poly(dimethylsiloxane) (PDMS)-based protein chip platform for dual detection of PRV gD and gE Abs, in which the viral proteins were printed onto the activated PDMS membrane by the spotter. The sensitivity of the dual-detection platform and its potential for clinical application were evaluated.

## Materials and methods

### Serum samples

PRV-negative and -positive pig serum samples identified by neutralization test and using the PRV/ADV gE Ab Test Kit (IDEXX, USA) were collected by Luoyang Putai Biotech Co., Ltd. The clinical pig serum samples (n = 270) that are negative of PRV gE Ab and neutralizing Ab and clinical pig serum samples (n = 1,056) that were randomly selected were collected by Luoyang Putai Biotech Co., Ltd. Forty serum samples from five pigs immunized with PRV-inactivated vaccine (HN1201-△gE) and 80 serum samples from 10 pigs nasally challenged with HN1201 (n = 5) and Fa (n = 5) (collected at 0, 3, 5, 6, 7, 9, 11, and 14 days post-challenge) were provided by the National Research Center for Veterinary Medicine. Positive pig serum samples of porcine circovirus type 2 (PCV2), porcine parvovirus (PPV), porcine reproductive and respiratory syndrome virus (PRRSV), classical swine fever virus (CSFV), porcine epidemic diarrhea virus (PEDV), porcine deltacoronavirus (PDCoV), FMDV serotype O (pig), FMDV serotype A (pig), and baculovirus were collected by Luoyang Putai Biotech Co., Ltd. Positive pig serum samples of African swine fever virus (ASFV) were purchased from the China Institute of Veterinary Drug Control. All the animal samples were collected according to the protocol approved by the Animal Care and Ethics Committee of National Research Center for Veterinary Medicine (Permit 20170012).

### Cells

Sf9 cells were cultured in the Sf-900™ III SFM medium (Gibco, USA). PK-15 cells were cultured in Dulbecco’s modified Eagle medium (DMEM) (Gibco) containing 4% fetal bovine serum (FBS) (Gibco, USA).

### Production of PRV gD and gE proteins

The PRV variant HN1201 (GenBank accession no. KP722022.1) isolated in 2012 has been described previously (Wu et al., 2013). We used the Bac-to-Bac^®^ TOPO^®^ Expression System (Invitrogen, USA) for protein expression. In the plasmid construction, we amplified the gD and gE genes (removal of the transmembrane and intracellular domains) of the PRV HN1201 strain using the primer pairs gD-F/gD-R-His and gE-F/gE-R-His (**Table 1**), respectively. After digestion with *Bam* HI and *Hind* III, the purified PCR fragments were ligated into the pFastBac I vector to construct the donor plasmids pFB-gD-His and pFB-gE-His. Afterward, the positive pFB-gD-His and pFB-gE-His were transformed into DH10Bac competent cells for constructing recombinant plasmids Bacmid-gD-His and Bacmid-gE-His, which were selected by gentamicin, blue/white colonies, and the PCR using the pUC/M13 primers (**Table 1**). To rescue the recombinant baculovirus rPRV-gD-His and rPRV-gE-His, the plasmids of Bacmid-gD-His and Bacmid-gE-His were transfected into Sf9 cells, respectively. To express the gD and gE proteins, Sf9 cells were infected with the recombinant PRV strains rPRV-gD-His and rPRV-gE-His at a multiplicity of infection (MOI) of 1.0, respectively. After infection, the supernatant of the Sf9 cells was harvested when the cells were found to have increased in diameter, then the gD and gE proteins were collected and purified by affinity chromatography of HisTrap™ HP (GE Healthcare, USA) and size exclusion chromatography of HiLoad^®^ 16/600 Superdex^®^ 200 pg (GE Healthcare).

**Table 1:** Primers used in this study.

Primers	Sequences (5’-3’)
gD-BamHI-F ^a^	CGGGATCCATGCTGCTCGCAGCGCTATTGGCGG
gD-His-HindIII-R ^b^	CCAAGCTTCTA ***GTGATGGTGATGGTGATG*** GCGGTGGCGCGAGACGCCCGGCG
gE-BamHI-F ^a^	CGGGATCCatgcggccctttctgctgcgcgc
gE-His-HindIII-R ^b^	CCAAGCTTCTA ***GTGATGGTGATGGTGATG*** GGGCGTCGTCCGGCCGTACGGGT
pUC/M13-F	CCCAGTCACGACGTTGTAAAACG
pUC/M13-R	AGCGGATAACAATTTCACACAGG

a the sequence of Bam HI was underlined.

b the sequence of Hind III was underlined, the sequence of 6×His tag was shown in bold and italic.

### Construction of nanomembrane-based protein chip platform

#### Membrane activation

The nanomembrane with PDMS brushes on the surface, formed through the surface-initiated polymerization by initiator integrated PDMS (iPMDS), was placed in an activation tank and then activated by incubating with the solution containing 1-(3-dimethylaminopropyl)-3-ethylcarbodiimide (EDC) and N-hydroxysulfosuccinimide (NHS); the surface of the nanomembrane should be completely immersed in the solution. After soaking for 30 min, the nanomembrane was washed with ultrapure water three times and blow-dried.

#### Protein spotting and the reaction procedure

**Figure 1:**
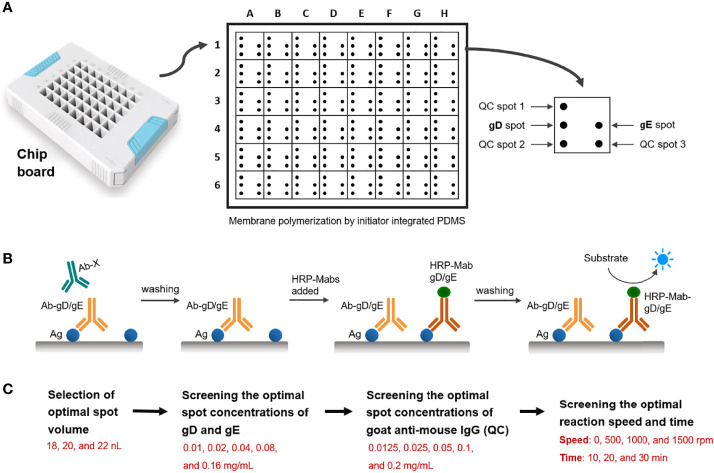
Schematic diagram of the design of the double Abs detection system. **(A)** Spotting design of detection points in the hole of the chip board. QC, quality control. **(B)** Schematic diagram of the steps of the blocking ELISA method for PRV gD and gE Abs detection. Ag: gD or gE coated in the membrane. Ab-gD/gE: PRV gD- or gE-specific Ab in serum samples. Ab-X: PRV gD- or gE-non-specific Ab in serum samples. HRP-Mab-gD/gE: HRP-labeled Mabs of PRV gD or gE in the blocking assay. **(C)** Screening process for the reaction conditions of the detection system, including the optimal spot volume, spot concentrations of gD, gE and goat anti-mouse IgG, and reaction speed and time.

According to the spotting design (**Figure 1A**), a spotter was used to prepare a dual-detection chip aimed at PRV gD and gE Abs, including three spots for quality control, one spot for gD, and one spot for gE. Microchip assay technology for Abs detection of PRV gD and gE is based on the blocking ELISA procedure (**Figure 1B**). The membrane surface was coated with gD/gE, then the gD/gE-specific Abs in the samples bound to gD/gE; after washing, the horseradish peroxidase (HRP)-labeled gD/gE specific Ab was added. If there is still unbound gD/gE on the membrane, after washing, the added substrate would show Ab in the samples; the high value indicated the less specific Abs in the sample to be tested. To prepare the working solutions for spotting the targeted protein, the purified PRV gD and gE proteins were diluted using the spotting diluent; the protein concentration was adjusted to 0.1 mg/ml for the selection of optimal spot volume in 18, 20, and 22 nanoliters (nl) (**Figure 1C**). The spotted antigens at the corresponding sites are linked to the surface of the nanomembrane by chemical covalent bonds. The optimal spot volume was determined by the highest positive/negative (N/P) ratio, which was calculated based on the gray values after incubation with the PRV positive and negative serum samples. The proteins as the control were spotted in the polyvinylidene fluoride (PVDF) membrane.

After determining the optimal spot volume in this system, we screened the optimal spot concentrations of gD, gE, and Goat Anti-Mouse IgG. In total, five dilution steps of viral proteins (0.01, 0.02, 0.04, 0.08, and 0.16 mg/ml) and five dilution steps of goat anti-mouse IgG (0.0125, 0.025, 0.05, 0.1, and 0.2 mg/ml) were analyzed (**Figure 1C**). By analyzing the PRV-positive and -negative serum samples, the optimal spot concentrations of gD and gE proteins were determined by the highest N/P ratio calculated based on the gray values, and the optimal spot concentration of goat anti-mouse IgG was determined based on the gray value closest to that of the PRV-negative serum sample.

As with the determination of the optimal conditions above, we analyzed the reaction speed (0, 500, 1,000, and 1,500 rpm) and reaction time (10, 20, and 30 min) in the system. The optimal conditions of reaction speed and reaction time were determined by the highest N/P ratios by analyzing the PRV-positive and -negative serum samples.

The environmental conditions for spotting were controlled at the temperatures of 20°C–25°C and the humidity of 40%–70%. Each test was performed at least in duplicate.

To determine the cutoff value, a total of 270 PRV gD- and gE-negative pig serum samples identified by neutralization assay (gD Ab) and a commercial kit (gE Ab) were used. The cutoff value was determined according to the distribution characteristics of the S/N (sample/negative) ratio data generated from the serum samples. The positive and negative cutoff values were settled as the mean of S/N value - 3 × standard deviation (SD) and the mean of S/N value - 2 × SD, respectively (Lardeux et al., 2016). In the chip method based on the blocking type ELISA, the lower S/N value represents a high target Ab level in the sample.

#### Chip assembly

The spotted nanomembrane was placed in the chip holder’s base then installed using an upper cover and clamping strips. After assembling, the chip was sealed with the blocking buffer of 1% BSA (200 µl/well). After blocking at 20°C–25°C for 1 h, the blocking buffer was discarded. The chip was dried at temperatures of 20°C–25°C and a humidity of 20% for 16–20 h. Afterward, the dried chip was vacuum-encapsulated into an aluminum foil bag with desiccant then stored at 2°C–8°C.

### Evaluation of chip detection system

#### Sensitivity

In total, we used 40 serum samples of HN1201-△gE strain-immunized pigs and 80 serum samples of PRV-challenged pigs to evaluate the sensitivity. The results of the gD and gE Abs tests were compared with those of the PRV neutralization test and a commercial PRV gE protein Ab test kit, respectively.

In the PRV neutralization test, briefly, the serum samples were inactivated at 56°C for 30 min and then twofold serially diluted with DMEM. After that, the diluted serum samples (50 µl) were mixed with an equal volume of HN1201 (200 TCID_50_) and incubated at 37°C for 1 h; then, the mixture samples were added to the PK-15 cell seeded wells (2 × 10^4^ cells per well) in the 96-well plates (four repetitions, 100 µl/well). After incubation at 37°C in an incubator containing 5% CO_2_ for 5 days, the cytopathic effect caused by PRV infection was recorded. The titers of PRV-specific NAbs were expressed as the reciprocal of the highest dilution at which infection of the PK-15 cells was inhibited in 50% of the culture wells. The detection of the PRV gE Ab is performed in accordance with the instructions of the commercial kit.

#### Specificity

We used the positive serum samples of ASFV, PCV2, PPV, PRRSV, CSFV (Zhang et al., 2020), PEDV, PDCoV, FMDV serotype O (pig), FMDV serotype A (pig) and baculovirus (5 samples for each virus) to test the reactivity of the protein chip kit. The detection procedure was according to the steps in the “Protein spotting and the reaction procedure” section.

### Clinical trial evaluation

In the evaluation of the clinical application, we used the protein chip to detect 1,056 pig serum samples collected clinically. Among these, 122 serum samples were randomly selected for neutralization assay and commercial gE Ab kit and then compared with the gD and gE test results of the protein chip detection, respectively.

### Statistical analysis

The frequency distribution was constructed using GraphPad Prism 8.0. The comparison of chip detection and commercial kit methods was analyzed by one-way ANOVA using GraphPad Prism 8.0. The results were presented as the mean ± SD, and *p* < 0.05 was considered statistically significant. In addition, kappa and 95% confidence interval were calculated at https://www.graphpad.com/quickcalcs/kappa1/.

## Results

### Purified PRV gD and gE proteins for membrane coating

**Figure 2:**
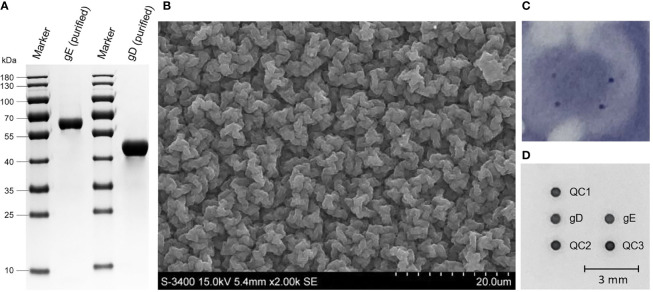
Expression of PRV gD and gE and the Ab detection based on the PDMS membrane. **(A)** SDS-PAGE analysis of the purified gD and gE proteins expressed in Sf9 cells after size exclusion chromatography. **(B)** Scanning electron microscopy of the PDMS membrane. **(C)** Abs detection results of gD and gE in the nitrocellulose membrane. **(D)** Abs detection results of gD and gE in the PDMS membrane. mm, millimeter.

The recombinant baculovirus rPRV-gD-His and rPRV-gE-His were rescued after transfection of Bacmid-gD-His and Bacmid-gE-His, respectively. For protein expression, Sf9 cells were infected with rPRV-gD-His and rPRV-gE-His at the MOI of 1.0. At 48 h after infection, the supernatants of the Sf9 cells were harvested and analyzed. The results of SDS-PAGE (**Figure 2A**) showed that the purified gD and gE proteins (molecular weight of about 45 and 62 kDa, respectively) could be observed. The purified gD and gE proteins were stored at the concentrations of 2.0 and 3.0 mg/ml, respectively.

### Determination of the optimum conditions for the chip detection system

The spotting results of nitrocellulose membranes showed the difficulty of standardizing the dots on the membrane surface; the digitized grayscale data were not suitable for acquisition (**Figure 2C**). In the PDMS membrane (**Figure 2B**), after selection by the highest N/P ratio, the optimal spotting volumes for PRV gD and gE proteins were settled as 20 nl, and the optimal spotting concentrations of gD and gE proteins were 0.04 and 0.02 mg/ml, respectively. The optimal concentration of goat anti-mouse IgG was 0.025 mg/ml. In addition, the reaction conditions of the detection system were determined as follows. 1) The serum to be tested is mixed with the diluent in a volume ratio of 1:1 (50 µl + 50 µl) and then incubated with shaking at 37°C for 20 min (1,000 rpm). 2) The incubation solution is discarded. 3) After washing with PBS’T, the HRP labeling reagent is added at 100 µl per well and then incubated with shaking at 37°C for 20 min (1,000 rpm). 4) The incubation solution is discarded. 5) After washing with PBS’T, the TMB (3,3′,5,5′-tetramethylbenzidine) substrate solution is added at 100 µl per well and then incubated with shaking at 37°C for 15 min (1,000 rpm). For results calculation, the S/N value = (the gray value of the detection point)/(the average gray value of the quality control points 1, 2, and 3). The microplate reader was used to automatically calculate the S/N value based on the gray values in the chip membrane, and then a test report was issued (**Figure 2D**).

### Determination standard of the chip detection system

**Figure 3:**
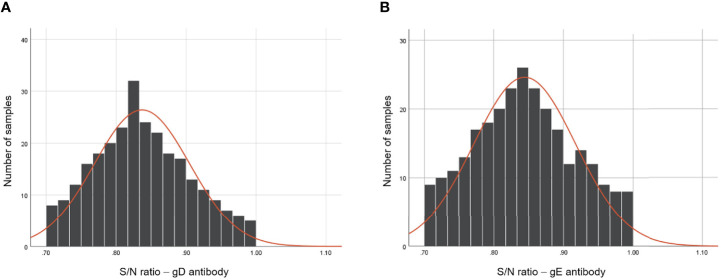
Frequency analysis of PRV gD and gE Abs-negative serum samples using the dual-detection chip platform. The S/N values of gD **(A)** and gE **(B)** Abs of 270 Ab-negative serum samples after the detection of the dual detection chip platform. The frequency distribution of the tested serum samples was constructed using GraphPad Prism 8.0.

Results from the S/N values of 270 clinically negative serum samples showed the typical frequency distributions in the histogram (**Figures 3A, B**) for gD and gE Ab testing. For gD, the positive and negative cutoff values were settled as the mean of S/N value - 3 × SD (0.633, ~0.6) and the mean of S/N value - 2 × SD (0.701, ~0.7), respectively. The determination standards of gD Ab were settled as follows: positive, S/N ≤0.6; suspicious, 0.6 < S/N ≤ 0.7; negative, S/N > 0.7. For gE, the positive and negative cutoff values were settled as the mean value of S/N value - 3 × SD (0.626, ~0.6) and the mean value of S/N value - 2 × SD (0.698, ~0.7), respectively. The determination standards of gE Ab were settled as follows: positive, S/N ≤ 0.6; suspicious: 0.6 < S/N ≤ 0.7; negative, S/N > 0.7.

### The sensitivity evaluation result of the chip detection system

**Figure 4:**
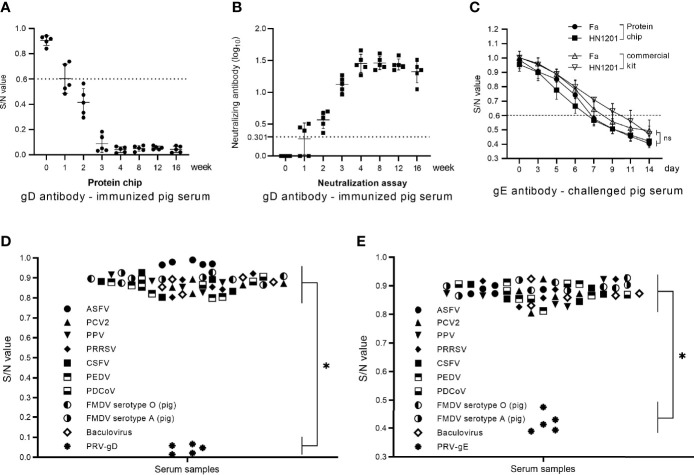
Sensitivity and specificity evaluation of the chip detection system in gD and gE Abs. **(A)** The gD Ab of immunized pig serum samples (0–16 wpi) tested by the protein chip detection system. **(B)** The gD Ab of immunized pig serum samples (0–16 wpi) tested by neutralization assay. **(C)** The gE Ab of challenged pig serum samples (0–14 dpc) tested by the protein chip detection system and commercial gE Ab kit. **(D)** Specificity assay of the chip detection system in gD Ab detection using the positive serum samples of ASFV, PCV2, PPV, PRRSV, CSFV, PEDV, PDCoV, FMDV serotype O (pig), FMDV serotype A (pig), baculovirus, and PRV-gD (n = 5). **(E)** Specificity assay of the chip detection system in gD Ab detection using the positive serum samples of ASFV, PCV2, PPV, PRRSV, CSFV, PEDV, PDCoV, FMDV serotype O (pig), FMDV serotype A (pig), baculovirus, and PRV-gE (n = 5). S/N value > 0.7 is considered negative. The comparison of chip detection and commercial kit methods was analyzed by one-way ANOVA using GraphPad Prism 8.0. ns, not significant. *p < 0.05.

For sensitivity evaluation of the chip detection system in the gD Ab test, we used the chip detection system and PRV neutralization test to detect the gD Ab of the serum samples from PRV-inactivated vaccine (HN1201-△gE strain)-immunized pigs. In the serum samples collected at 1 week post-immunization (wpi), both the chip detection system and the neutralization test showed a positive rate of 3/5. At 2 wpi, the result of both two methods showed a positive rate of 5/5. So, the two methods showed an agreement of 100% in the sensitivity evaluation. Moreover, a higher level of gD Ab could be detected at 4 wpi and then maintained at the higher level until 16 wpi (**Figure 4A**). The sensitivity of this chip detection system for gD Ab detection was consistent with the neutralization test results (**Figure 4B**).

For a sensitivity evaluation of the chip detection system in the gE Ab test, we used the chip detection system and the commercial PRV gE Ab detection kit to detect the gE Ab of the 80 serum samples from HN1201-challenged pigs. The chip detection system showed that the gE seroconversion in Fa strain-challenged pigs could be detected at 7 days post-challenge (dpc), and all the samples turned positive at 9 dpc, while in HN1201 strain-challenged pigs, the serum samples showed a gE seroconversion at 6 dpc, and all turned positive at 9 dpc. In the assay using the commercial PRV gE Ab detection kit, gE seroconversion was detected at 7 dpc, and all the samples turned positive at 11 dpc. In contrast, in HN1201 strain-challenged pigs, the serum samples showed a gE seroconversion at 9 dpc, and all turned positive at 14 dpc (**Figure 4C**). The results showed better sensitivity of the chip detection system in the gE Ab test than that of the commercial PRV gE Ab detection kit.

### Evaluation results of the chip detection system in specificity and reproducibility

For specificity assay, we used the chip detection system to detect the positive serum samples of ASFV, PCV2, PPV, PRRSV, CSFV, PEDV, PDCoV, FMDV serotype O (pig), FMDV serotype A (pig), and baculovirus, respectively. The results showed that all the serum samples were negative for gD and gE Abs (**Figures 4D, E**), indicating a good specificity of the chip detection system.

### Ab assessment results of clinical serum

We used the chip detection system to detect 1,056 clinically collected pig serum samples, and the results showed that the gD Ab-positive and -negative serum samples were 889 (84.2%) and 167 (15.8%), respectively. For gE Ab detection, the positive and negative serum samples were 287 (27.2%) and 769 (72.8%), respectively. To explore the coincidence of the test results, a total of 122 serum samples, analyzed by the neutralization test and the commercial PRV gE Ab detection kit, were selected for further study. The results (**Table 2**) showed a coincidence rate of 100% (122/122) in the gD Ab test between the neutralization assay and the chip detection system, and a coincidence rate of 97.5% (119/122) in gE Ab between the chip detection system and the commercial PRV gE Ab detection kit.

**Table 2:** Comparison of the chip detection system, neutralization assay and commercial PRV gE antibody detection kit for gD and gE antibody detection of clinical samples.

		Neutralization assay (gD)				Commercial kit (gE)		
		+ ^a^	- ^b^	Total		+	-	Total
Chip detection system	+	77	0	77		37	3	40
	–	0	45	45		0	82	82
	Total	77	45	122		37	85	122
Concordance rate		100% (122/122)				97.5% (119/122)		
Kappa ^c^		1.000				0.943		
95% confidence interval ^c^		1.000 - 1.000				0.880 – 1.000		

a “+”, positive.

b “-”, negative.

c Kappa and the 95% confidence interval was calculated at https://www.graphpad.com/quickcalcs/kappa1/.

## Discussion

We used the PDMS-coated nanomembrane as the reaction carrier of the protein chip detection system and then immobilized the antigen on the surface of the PDMS-coated nanomembrane in the form of microdots by an automatic spotter to form a microarray. We chose the PDMS-coated nanomembrane as the carrier of the protein detection chip due to its strong resistance to non-specific adsorption. For hydrophobic materials, PDMS brushes created by a “grafting-to” process are shown to be suitable coatings to increase the system’s stability (Green and Mewis, 2006). Here, the functional groups are covalently linked on the nanomembrane surface by iPMDS through surface-initiated polymerization (Harabagiu et al., 2011; Zhang et al., 2011; Koch et al., 2020); thus, a large number of carboxyl groups are exposed on the surface to form a polymer layer to resist protein adsorption, which we called “0 + X” nanomembrane.

The “0 + X” nanomembrane was activated by EDC and NHS, resulting in high molecularly active carboxyl groups being converted into intermediate lipid structures that can be linked to protein amino groups (Madler et al., 2009; Totaro et al., 2016). At this time, the spotted antigens at the corresponding sites are linked to the surface of the nanomembrane by chemical covalent bonds. After 4 h of activation, the ester of the unbound protein was converted into a carboxyl group, and the anti-protein adsorption function was restored.

In the “0+X” nanomembrane, “0” means that the area outside the target protein has no background interference induced by the anti-protein adsorption function on the membrane surface, which could effectively avoid the background interference caused by the non-specific adsorption on the traditional nitrocellulose membrane (**Figure 2C**), indicating the improved sensitivity and specificity. The “X” means that any target protein with an amino group can be linked during membrane activation; any spotting site can be selected according to the experimental requirement, and even the combination of different antigens or different antigen concentrations, providing the possibility for the product development of multiple antigens or Abs. Compared with the physical adsorption of the antigens in the ELISA, the covalent bonds in the “0+X” nanomembrane are more stable, and the high-throughput detection could be realized.

Gene-deleted live vaccines like Bartha-K61 accompanied by differential serological tests were effective in PR control worldwide (Mettenleiter, 1994; Luo et al., 2014). PRV gE was critical for PRV virulence (Zhao et al., 2020), and gD is a most efficacious virus-neutralizing Ab target of PRV (Eloit et al., 1988; Mukamoto et al., 1991). The emerging virulent PRV strains have caused severe PR in the vaccinated pigs in China since late 2011 (Yu et al., 2014; Gu et al., 2015), which showed the urgency to develop a more rapid and accurate PRV detection method that is suitable for clinical application for the assessment of neutralizing Ab and differentiation between vaccine and wild-type strains. So, we applied the “0+X” nanomembrane-based chip detection platform to the detection of PRV gD and gE Abs.

In this study, we solidified PRV gD and gE proteins in a single well to achieve the dual detection of two Abs. Compared with the ELISA method and fluorescent-encoded microsphere-based assay for PRV Ab detection (Ji et al., 2020), this chip detection platform is more convenient due to the automatic production and detection procedures, providing a time-saving choice for Ab detection with high objectivity. In addition, the dual detection of gD and gE proteins in a single well showed high clinical applicability to evaluate the immune effect of the PRV vaccine and distinguish PRV vaccine and wild-type strains. Moreover, based on the “0+X” nanomembrane, three or more target antigens could be designed for solid-phase spotting, even for detecting multiple pathogens.

The sensitivity and specificity of the chip detection platform for detecting gD Ab were consistent with that of the neutralization test. For gE Ab detection, the chip detection platform showed higher specificity and sensitivity than the commercial kits. This provides an accurate and specific detection platform for dual-monitoring PRV Ab and differentiating wild-type PRV infection from the vaccinated response in animals.

## Data availability statement

The datasets presented in this study can be found in online repositories. The names of the repository/repositories and accession number(s) can be found in the article/supplementary material.

## Ethics statement

The animal study was reviewed and approved by Research Ethics Committee of the National Research Center for Veterinary Medicine.

## Author contributions

JP, YL, and JD conceived and designed the research. JP, YL, TW, JC, and LH conducted the experiments. YL, BH, JC, WP, and JD analyzed the data. JP, YL, BH, and KT carried out additional analyses and finalized the manuscript. All authors contributed to the article and approved the submitted version.

## Funding

This study was supported by the project of R&D and industrialization of genetically engineered vaccines for swine pseudorabies, swine ring, and Mycoplasma hyopneumoniae (201200211200).

## Conflict of interest

Authors JP, JC, LH, WP, and JD are employed by Luoyang Putai Biotech Co., Ltd. Author YL is employed by Luoyang Zhongke Biochip Technology Co., Ltd.

The remaining authors declare that the research was conducted in the absence of any commercial or financial relationships that could be construed as a potential conflict of interest.

## Publisher’s note

All claims expressed in this article are solely those of the authors and do not necessarily represent those of their affiliated organizations, or those of the publisher, the editors and the reviewers. Any product that may be evaluated in this article, or claim that may be made by its manufacturer, is not guaranteed or endorsed by the publisher.
